# Evaluating cutpoints for the MHI-5 and MCS using the GHQ-12: a comparison of five different methods

**DOI:** 10.1186/1471-244X-8-10

**Published:** 2008-02-19

**Authors:** Mark J Kelly, Frank D Dunstan, Keith Lloyd, David L Fone

**Affiliations:** 1 Dept. of Primary Care and Public Health, Centre for Health Sciences Research, School of Medicine, Cardiff University, Heath Park, Cardiff, CF14 4YS, UK; 2 Centre for Health Information, Research and Evaluation, School of Medicine, Swansea University, UK

## Abstract

**Background:**

The Mental Health Inventory (MHI-5) and the Mental Health Component Summary score (MCS) derived from the Short Form 36 (SF-36) instrument are well validated and reliable scales. A drawback of their construction is that neither has a clinically validated cutpoint to define a case of common mental disorder (CMD). This paper aims to produce cutpoints for the MHI-5 and MCS by comparison with the General Health Questionnaire (GHQ-12).

**Methods:**

Data were analysed from wave 9 of the British Household Panel Survey (2000), providing a sample size of 14,669 individuals. Receiver Operating Characteristic (ROC) curves were used to compare the scales and define cutpoints for the MHI-5 and MCS, using the following optimisation criteria: the Youden Index, the point closest to (0,1) on the ROC curve, minimising the misclassification rate, the minimax method, and prevalence matching.

**Results:**

For the MHI-5, the Youden Index and the (0,1) methods both gave a cutpoint of 76, minimising the misclassification rate gave a cutpoint of 60 and the minimax method and prevalence matching gave a cutpoint of 68. For the MCS, the Youden Index and the (0,1) methods gave cutpoints of 51.7 and 52.1 respectively, minimising the error rate gave a cutpoint of 44.8 and both the minimax method and prevalence matching gave a cutpoint of 48.9. The correlation between the MHI-5 and the MCS was 0.88.

**Conclusion:**

The Youden Index and (0,1) methods are most suitable for determining a cutpoint for the MHI-5, since they are least dependent on population prevalence. The choice of method is dependent on the intended application. The MHI-5 performs remarkably well against the longer MCS.

## Background

The common mental disorders of anxiety and depression (CMD) are leading causes of morbidity and disability and constitute a major public health burden [1]. The CMDs are most commonly measured in population studies using the General Health Questionnaire (GHQ-12) [2]. Another frequently used scale is the Mental Health Inventory (MHI-5) [3] which is included in the Short Form 36 (SF-36). The MHI-5 is a well validated and reliable measure of mental health status [4], but an important limitation of its use is that it was not developed with a validated cutpoint to define a case of CMD. The SF-36 can also be used to construct the Mental Health Component Summary score (MCS) which is another measure of mental health status that is widely used in population surveys but has no clinically validated cutpoint [5]. In this paper we aim to derive generalisable cutpoints for the MHI-5 and MCS using the GHQ-12 as a gold standard, using five different optimisation criteria.

## Methods

### Dataset

Data from wave 9 of the British Household Panel Survey (BHPS) [6] were used in this analysis. The BHPS is a longitudinal study carried out in England, Scotland and Wales (in wave 9). The first wave of the BHPS was carried out in 1991 with a nationally representative sample of 5,500 households. The BHPS follows households through time, with an annual interview of every member of the household aged 16 and over. Individuals interviewed in the first sample who subsequently set up their own household continued to participate in the survey, as well as every individual in the new household. All waves of the BHPS include the GHQ-12, but wave 9 of the study (2000) also included the SF-36 version 1. There is complete information on both of these instruments for all 14,669 individuals in the dataset. Of those present at wave 8, 83.4% were successfully followed up at wave 9.

### Mental Health Measures

The GHQ-12 comprises twelve questions, each with a set of Likert scale responses which score the question as 0, 1, 2 or 3. There are two ways of scoring the GHQ-12. Either the sum of these responses is used to provide a score ranging between 0 and 36 or alternatively, the response to each question is deemed positive if it is greater than one and the number of positives provides the score. This results in a score between 0 and 12 for each individual. This latter method is used in this study. Different studies use different cutpoints between 2 and 4 to define a case of common mental disorder. In this paper we use the most widely accepted convention of a score of three or more defined as a case [7]. The SF-36 version 1 consists of eight subscales measuring Physical Functioning, Role Physical, Bodily Pain, General Health, Vitality, Social Functioning, Role Emotional and Mental Health. The MHI-5 comprises five questions. There are six possible responses to the questions, scored between 1 and 6. The score for each individual therefore ranges between 5 and 30. This is then transformed into a variable ranging from 0–100 using a standard linear transformation [5]. A different mental health score, which incorporates all eight subscales of the SF-36, called the Mental Health Component Summary (MCS) can also be constructed. It was calculated in the standard way [5], using UK norms [8] and factor loadings [9].

On both the MHI-5 and the MCS high scores indicate good mental health, unlike the GHQ-12. Both the MHI-5 and the GHQ-12 scales have discrete distributions. The GHQ-12 takes on only 13 different values, while the MHI-5 produces only 26 different values. The MCS is a continuous variable, with 11,003 different values being calculated for the 14,669 individuals in the BHPS dataset.

### Statistical Methods

#### Sensitivity and Specificity

In order to identify a cutpoint for any new measure, it needs to be compared to another scale which can classify people as a case or a non-case. Ideally, this scale would be a gold standard and would produce no misclassifications. In the field of mental health the acknowledged gold standard is a standardised interview. A well validated scale, such as the GHQ-12, with an associated cutpoint to distinguish cases from non-cases is a good alternative. The GHQ-12 classifies each individual in the dataset as a case or a non-case. Our aim is to find the cutpoints on the MHI-5 and MCS that imitate the GHQ-12 cutpoint as closely as possible. Individuals with mental health scores less than or equal to the cutpoint on the MHI-5 or MCS will be defined as cases. The evaluation of a cutpoint involves the twin concepts of sensitivity and specificity. The sensitivity of a test is the probability of a case testing positive (i.e. a true positive). The specificity of a test is the probability of a non-case testing negative (i.e. a true negative). Clearly a good test has a large sensitivity, but a test which automatically classifies everyone as a case has a sensitivity of one (the maximum possible), even though it is completely uninformative. There is a trade-off to be made, then, between sensitivity and specificity. As the cutpoint is decreased, the sensitivity decreases, while the specificity increases.

#### Receiver Operating Characteristic Curves

For each possible cutpoint on the measure under investigation there is an associated sensitivity and specificity. These can be summarised using a receiver operating characteristic (ROC) curve. A ROC curve plots the sensitivity (i.e. true positive rate) on the y-axis against one minus the specificity (i.e. false positive rate) on the x-axis. Each point on the curve represents a different cutpoint on the new measure. A diagonal line at 45 degrees, known as the line of chance, would result from a test which allocated subjects randomly.

#### Optimisation Criteria

There are several approaches to choosing a cutpoint on a ROC curve. Five of these will be investigated in this study. Each method focuses on optimising a different criterion and so may produce a different cutpoint. The five methods are: 1. the Youden Index [10], 2. the point closest to the upper left corner, coordinates (0,1), as used by Holmes [11] 3. the misclassification rate, 4: the minimax method [12] and 5. prevalence matching, as used by Hoeymans et al [13]. Only the first two have a graphical interpretation on the ROC curve.

##### Youden Index

In general, a good cutpoint is one which produces both a large sensitivity and a large specificity. An intuitive method, therefore, is to maximise the sum of the sensitivity and specificity, S, i.e. satisfy equation 1

S = *max*(Sensitivity + Specificity)

This approach assumes that sensitivity and specificity are equally important. This is exactly equivalent to the Youden index, shown in equation 2, since subtracting a constant does not affect the optimal cutpoint. This can be interpreted as choosing the point on the ROC curve with the largest vertical distance from the line of chance.

*J *= *max*(Sensitivity + Specificity - 1)

##### Shortest distance to upper left corner

The second optimisation method investigated in this paper is to choose the cutpoint associated with the point on the ROC curve closest to the upper left corner. This entails finding the cutpoint which minimises *d *in equation 3. This method also places equal weight on the sensitivity and specificity.

$$d=\sqrt{{\left(1-Sensitivity\right)}^{2}+{\left(1-Specificity\right)}^{2}}$$

**Figure 1:**
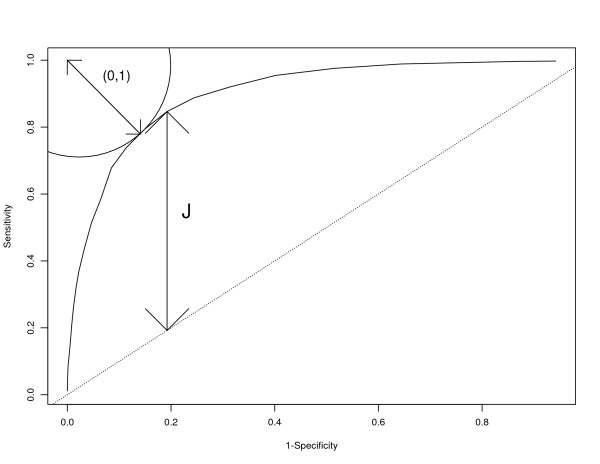
**Graphical illustration of the Youden Index (J) and the (0,1) criterion**. 1. (0,1) refers to the minimum distance between the point (0,1) and the ROC curve. 2. J refers to the Youden Index in equation 2.

The rationale behind this is that a perfect ROC curve would pass through the point (0,1) (i.e. *Sensitivity *= 1 &*Specificity *= 1 for some cutpoint). Selecting the point on the curve which is closest to this point of perfection is one way to choose a cutpoint. The Youden index and the (0,1) criterion are illustrated in Figure 1.

##### Misclassification rate

Alternatively, the misclassification rate could be minimised. For this we define the false positive rate (FPR) to be

FPR = (Non-case Prevalence)×(1-Specificity)

and the false negative rate (FNR) to be

FNR = (Case Prevalence)×(1-Sensitivity)

and it is the sum of these two terms that is minimised. This essentially gives weights to the sensitivity and specificity based on the prevalence of cases. If the population has a very low prevalence of cases, then more weight would be given to specificity. If the prevalence is high, then sensitivity takes precedence. This presupposes that the penalty incurred for a false positive is equal to that incurred for a false negative. If this does not hold, the sum can be weighted according to the penalties incurred for false positives and negatives, i.e. minimise

*θ*×(1-Sensitivity) + (1 - *θ*)×(1-Specificity)

where *θ *is the weight attached to the sensitivity. Choosing this weight may not be straightforward. For instance, in this study it is difficult to compare the consequences of both types of misclassification. Expression 6 is equivalent to equations 1 and 2 with *θ *set to 0.5, and equivalent to prevalence matching (described below) with *θ *set to the population prevalence.

##### Minimax Criterion

The minimax criterion involves minimising the frequency of the most common error. In a two by two classification table, this is equivalent to minimising the maximum of the off-diagonal elements.

This involves minimising *M *in equation 7.

*M *= *max*(FPR, FNR)

This is similar to minimising the misclassification rate, except instead of the sum of FPR and FNR being minimised, the maximum of the two terms is minimised.

##### Prevalence Matching

The final optimisation criterion we consider is to choose a cutpoint which results in the proportion of the screened population classified as positives (or cases) being closest to the gold standard case prevalence. Those classed as positives comprise both true and false positives and so expression 8 is minimised, where the True Positive Rate (TPR) is the sensitivity multiplied by the case prevalence.

|TPR + FPR - *P*(Case)|

It can be shown that in the continuous case (i.e. where the new measure is capable of infinite subdivision) this method is equivalent to the minimax method. In discrete cases, they will produce very similar results. It is important to clarify at this point, that unlike other studies which employ ROC curves, the area under the curve is not a meaningful criterion to use here. The area under the curve summarises the performance of an entire measure across all cutpoints. It is appropriate when two new measures are being compared against a gold standard in order to determine which of the new measures performs most similarly to the gold standard. It cannot, however, be used to determine an optimum cutpoint on a scale.

Since the method uses the same dataset both to define cutpoints and assess the performance of those cutpoints, there is the possibility of overestimating the sensitivity and specificity. This potential source of bias is investigated by repeating the analysis using 75% of the dataset (randomly selected), and then assessing the performance of the cutpoints produced on the remaining 25% of the dataset.

## Results

### MHI-5 Results

**Figure 2:**
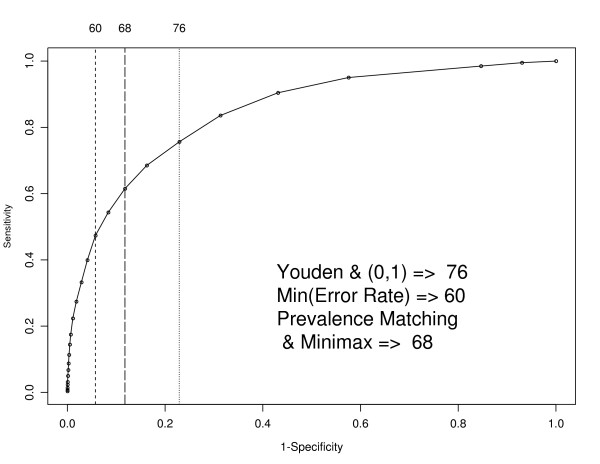
**MHI-5 ROC curve using a GHQ caseness criterion of 3 or more**. 1. ROC curve based on a GHQ-12 caseness criterion of 3 or more. Vertical lines indicate the optimum cutpoints using the five different optimisation criteria.

First consider the MHI-5. Maximising the Youden index leads to a cutpoint of 76 (a case of common mental disorder is defined by a score of less than or equal to 76) for the MHI-5. Using the shortest distance from (0,1) criterion the optimal cutpoint is also 76. In general, these two optimisation methods will not always give the same cutpoint though the discrete nature of both scales means that in practice they often will. Using the sample prevalence of 25.3% (according to the GHQ-12) to weight the sum of the sensitivity and specificity (thereby minimising the error rate) the corresponding cutpoint is 60. Using the prevalence matching method of choosing a cutpoint the optimal cutpoint is 68. This produces a case prevalence of 24.4%, which is the closest to the GHQ-12 case prevalence of 25.3%. The minimax method yields the same cutpoint as prevalence matching. The correlation between the GHQ-12 and the MHI-5 is high (-0.65). Figure 2 shows the points on the ROC curve corresponding to each of the cutpoints produced by the different optimisation criteria.

### MCS Results

**Figure 3:**
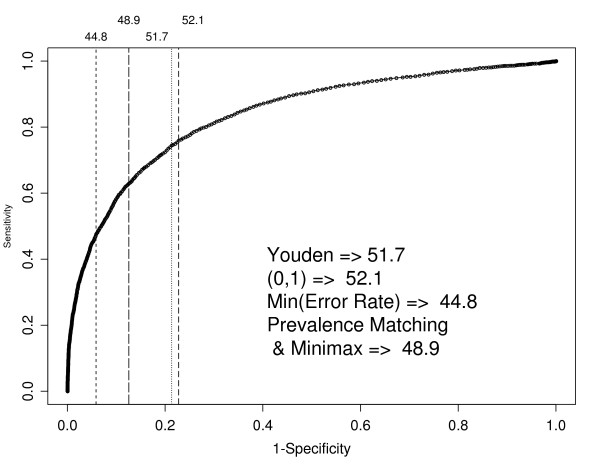
**MCS ROC curve using a GHQ caseness criterion of 3 or more**. 1. ROC curve based on a GHQ-12 caseness criterion of 3 or more. Vertical lines indicate the optimum cutpoints using the five different optimisation criteria.

Next, we examine the MCS. The Youden index and the (0,1) methods produce very similar cutpoints of 51.7 and 52.1, respectively. Minimising the error rate produces a cutpoint of 44.8 while both prevalence matching and the minimax method indicate a cutpoint of 48.9. Table 1 summarises the results and illustrates the trade off that must be made between sensitivity and specificity. Figure 3 shows the points on the ROC curve corresponding to each of the cutpoints produced by the different optimisation criteria. The correlation between the MCS and GHQ-12 is the same as for the MHI-5 at -0.65.

**Table 1:** MHI-5 and MCS cutpoints and associated test characteristics for five optimisation criteria

**Scale**	**Optimisation Criterion**	**Cutpoint**	**Sensitivity**	**Specificity**	**Positivity^**1 **^Rate**	**Error Rate^**2 **^%**
MHI-5	Youden Index	76	0.756	0.771	0.362	23.3
	(0,1)^3^	76	0.756	0.771	0.362	23.3
	Misclassification Rate	60	0.473	0.943	0.163	17.6
	Minimax method	68	0.615	0.882	0.244	18.5
	Prevalence Matching	68	0.615	0.882	0.244	18.5

MCS	Youden Index	51.7	0.745	0.787	0.348	22.4
	(0,1)	52.1	0.759	0.772	0.362	23.1
	Misclassification Rate	44.8	0.476	0.941	0.164	17.6
	Minimax method	48.9	0.630	0.874	0.253	18.8
	Prevalence Matching	48.9	0.630	0.874	0.253	18.8

^1^Positivity rate refers to the proportion of the sample defined to be a case using each cutpoint.

^2^Error rate refers to the proportion of the sample classified differently to the GHQ-12. This comprises both false negatives and false positives.

^3^(0,1) refers to the criterion which minimises the distance between the point (0,1) and the ROC curve

### Assessment of bias

Using three-quarters of the data to derive cutpoints resulted in no change of optimum cutpoints for the MHI-5. When these were applied to the unused 25% of the data in order to assess their performance no systematic bias was observed, with the sensitivity and specificity for each cutpoint being equally likely to increase as decrease. The situation was similar for the MCS, with most of the optimisation criteria producing identical cutpoints to those produced by the full dataset (the only method which produced a slightly different cutpoint was the minimising the misclassification method, which went from 44.8 to 45.1). Again, when these cutpoints were applied to the unused 25% of the data, they produced sensitivities and specificities very close to those reported for the full dataset.

### Comparison of the MHI-5 and the MCS

**Figure 4:**
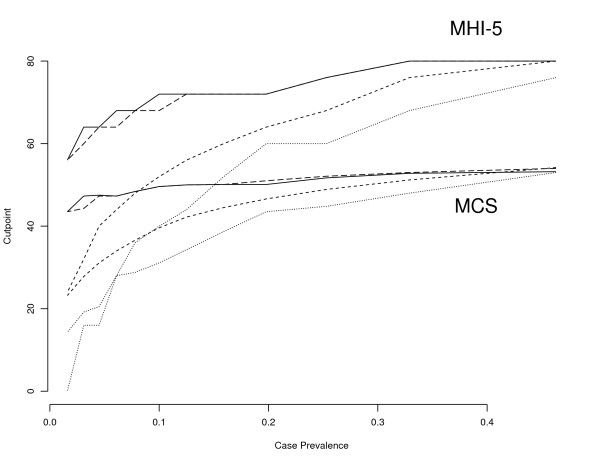
**Relationship between prevalence and MHI-5 and MCS cutpoints for four optimisation methods**. 1. Case prevalence is altered by varying the cutpoint used to define caseness on the GHQ-12 from 1 to 12. 2. Solid line denotes the Youden Index. 3. Dashed line denotes the (0,1) method. 4. Dotted line denotes the minimising the error rate method. 5. Dashed and dotted line denotes the prevalence matching method. 6. The minimax method is excluded since it is predominantly coincidental with the prevalence matching method.

It is also worth noting that the shorter MHI-5 performs remarkably similar to the longer MCS, with the correlation between the two being 0.88. Table 1 shows that the error rates produced by the five optimisation methods are very similar, for the two scales. Table 1 shows that the MCS is only marginally more efficient at discriminating cases of CMD than is the MHI-5, despite employing over seven times as many questions. Figure 4 illustrates how the cutpoints on the MHI-5 and the MCS vary with GHQ-12 case prevalence (the GHQ-12 case prevalence was varied by changing the cutpoint on the GHQ-12).

## Discussion

### Main Findings

For the MHI-5 the five methods produce three distinct cutpoints. Both graphical approaches (the Youden Index and the point closest to the upper left corner) produce a cutpoint of 76. Prevalence matching and the minimax method both indicate that 68 is the optimal cutpoint, while minimising the misclassification rate provides an optimal cutpoint of 60. The five methods produced similar results for the MCS with the two graphical approaches producing cutpoints of 51.7 and 52.1. Both prevalence matching and the minimax method gave an MCS cutpoint of 48.9 and minimising the missclassification rate produced an MCS cutpoint of 44.8. It is important to point out that the reason the prevalence matching, minimax, and minimising the misclassification methods give lower cutpoints than the Youden or (0,1) criteria is due to the fact that the case prevalence is less than 50% (25.3%). If the case prevalence were greater than 50% this situation would be reversed and the three aforementioned methods would give cutpoints greater than the Youden or (0,1) criteria.

The relationship between the optimum cutpoint and the population case prevalence for the five optimisation methods and for both the MHI-5 and the MCS was investigated in Figure 4 (the minimax method was excluded since it was largely coincidental with prevalence matching). For the minimising the error rate and prevalence matching methods the optimal cutpoint varies greatly with population prevalence, while the Youden index and (0,1) methods are relatively independent of population prevalence. This is the case for both scales. This invariance under different population prevalences is a property that is extremely useful for studies that span large and heterogeneous areas, such as international comparisons. Both methods also have intuitive interpretations as described earlier, and so there is very little to choose between them.

When the misclassification rate was minimised there was still a error rate of 17.6% for both the MHI-5 and MCS, which may imply that they measure slightly different constructs to the GHQ-12. This finding is echoed by Hoeymans et al [13] who noted that the MHI-5 was uncorrelated with age, whereas older age groups scored higher on the GHQ-12 (indicating worse mental health). Weinstein et al [14] drew attention to the fact that the comparative nature of the GHQ-12 response choices is not conducive to detecting chronic disorders. A subject suffering from chronic anxiety disorder may well answer the question "Have you recently lost much sleep over worry?" with the response choice "no more than usual", if their condition is a long-standing one. The MHI-5 and MCS avoid this problem by employing less comparative response choices. Another explanation for the lack of complete agreement between the GHQ-12 and the two SF-36 mental health measures is that they were designed differently. The MHI-5 includes one or more questions on each of the following mental health dimensions: anxiety, depression, loss of behavioural/emotional control and psychological well-being [3], while the MCS is a weighted sum of all eight health dimensions of the SF-36. The GHQ-12 on the other hand includes items on depression, anxiety, social performance and somatic complaints [2]. However, the high correlations between the GHQ-12 and both the MHI-5 and the MCS indicate that, despite these differences, the three scales perform very similarly. This can be seen in Table 1 where the five optimisation methods produce cutpoints on both scales with very similar properties in terms of sensitivity, specificity, positivity rate and error rate. As mentioned previously the correlation between the MHI-5 and the MCS is high at 0.88.

More generally, this study has found that the minimax method and prevalence matching methods give very similar results. Indeed, in this study they produce identical cutpoints. This is not a coincidence, as the two criteria become equivalent if the scale in question is continuous (and the probability of caseness is calculated from the same dataset).

Investigators should give careful consideration to which of these cutpoints is most appropriate for their study, since selecting which criterion should be optimised depends primarily on the intended application of the resulting cutpoint. For instance, a study whose primary goal is to identify cases in a given locality might do well to minimise the misclassification rate. However, a study interested in comparing CMD internationally should consider utilising the Youden Index or the (0,1) method, as these methods are most appropriate when the study area encompasses regions with different case prevalences. Prevalence matching has the advantage of simplicity but will inevitably lead to different cutpoints in different populations. The minimax method approximates to prevalence matching when the scale in question is continuous.

### Comparison with Previous Studies

One study of 7,359 adults representative of the Dutch general population used the GHQ-12 to derive a MHI-5 cutpoint using the prevalence matching method [13]. They used a less severe CMD case criterion of two or more on the GHQ-12, giving a case prevalence of 22.8%. The MHI-5 cutpoint which matched this prevalence most closely was 72, resulting in a case prevalence of 20.6%. To illustrate how this approach can lead to different results in different populations, we carried out the equivalent procedure in the BHPS dataset. Using a GHQ-12 caseness criterion of two or more classifies 32.9% of the dataset as cases. The MHI-5 cutpoint which best matches this prevalence is 76 (providing a case prevalence of 36.2%).

One small study compared four psychiatric case-finding instruments in 69 patients presenting to general practice in Wales and chose cutpoints which provided an undefined "similar sensitivity and specificity values for each instrument" [15]. The Revised Clinical Interview Schedule was used to define a case of CMD. This study identified an MHI-5 cutpoint quoted as 71/72.

A report published in Dutch compared the MHI-5 with the GHQ-12 in order to ascertain a cutpoint [16]. They sampled 7,065 independently living individuals aged 18 to 64 from the general population. A score of two or more on the GHQ-12 was used to define caseness, which classified 24.4% of the population as a case. They used the Youden Index and prevalence matching methods. The Youden Index indicated an MHI-5 cutpoint of 72, leading to a case prevalence of 22.8%. The Composite International Diagnostic Interview (CIDI) was used to determine whether individuals suffered from any of the following disorders: depression, bipolar disorder, dysthymia, panic disorder, agoraphobia, specific phobia, social phobia, generalised anxiety disorder, obsessive compulsive disorder, schizophrenia, anorexia and bulimia. The percentage of the population diagnosed with at least one of these disorders was found to be 12.2%. The MHI-5 cutpoint which matched this prevalence most closely was 60, producing a case prevalence of 11.2%.

Three other studies have defined a cutpoint by comparing MHI-5 scores with a range of different validated clinical interview schedules. These are summarised in turn below. The wide range of cutpoints found reflects the wide variety in sample sizes, study settings and outcomes of interest.

A study of 95 non-psychiatric patients who were HIV seropositive used the Structured Clinical Interview for DSM-III-R (SCID-NP-HIV) psychiatric disorders [11] and found a cutpoint of 52 using the (0,1) method. This study was investigating more severe disorders than the CMD and so produced a very low cutpoint. Applying this cutpoint to the BHPS dataset would identify only 8.3% of the individuals as cases. A study of 4,036 German nationals resident in an area of approximately 50 km in diameter surrounding Lubeck used the Munich Composite International Diagnostic Interview (M-CIDI) and found a cutpoint of 65 [17]. This study used the (0,1) method. This low cutpoint can be attributed to the fact that the M-CIDI is used to diagnose DSM-IV Axis 1 psychiatric disorders which are more extreme conditions than the common mental disorders.

Another study investigated the validity of the MHI-5 for assessing major depression using 1,444 functionally impaired, community dwelling elderly Americans. The gold standard against which the MHI-5 was compared was the MINI-International Neuro-Psychiatric Interview Major Depressive Episode (MINI-MDE) module. The Youden index optimisation criterion produced a cutpoint quoted as 59/60. Again, the study focussed on major depression and so produced a lower cutpoint than the cutpoint of 80 indicated by our paper.

A Norwegian study used the MHI-5 as the gold standard to define cutpoints for a different measure [18]. Postal questionnaire surveys with MHI-5 information were returned by 6865 (70.5% response rate) individuals and cutpoints of 52 and 56 were used successively.

To our knowledge no study has attempted to identify a cutpoint of the MCS.

### Strengths and Limitations of the Study

This study compares two measures derived from a questionnaire that is frequently employed in population research [18], using a large, representative sample of the UK population and five different optimisation criteria. The main strength of our study is that the sample size used is nearly twice that of the next largest study. So, while it is well known that optimal cutpoints vary as a function of the population being investigated, severity of caseness, case prevalence and gold standard employed, this study compares the cutpoints derived from five optimisation criteria, for the MHI-5 and the MCS. This facilitates an objective and comprehensive assessment of the best cutpoint for use in different studies.

A criticism of the approach adopted in this paper regards the use of the GHQ-12 as the comparative gold standard. In ROC curve analysis, a measure is supposed to be compared to a gold standard which can categorise the sample without error. In the field of mental health a standardised interview schedule is considered the gold standard and would be preferable to using the GHQ-12. A scale such as the GHQ-12 is likely to have lower sensitivity and specificity than a standardised interview schedule and this may affect the resulting cutpoints. Unfortunately, the BHPS did not administer a standardised interview schedule. A disadvantage to using a standardised interview schedule is that it is resource intensive, limiting the sample size achievable. Administering the GHQ-12 is, by comparison, inexpensive and efficient. Also, it can be argued that the GHQ-12 is a well validated and reliable scale, with validated cutpoints, and as such is a reasonable instrument against which to measure other scales.

A further potential criticism concerns the crude nature of cutpoints resulting in the loss of information in the variable being dichotomised. One method which seeks to avoid this problem is to use stratum-specific likelihood ratios (SSLRs) [19], defined as the ratio of the probability of a given test result when the disease is present and the probability of the same test result when the disease is absent. Instead of plotting these against one another as in a ROC curve, the SSLR approach examines the ratio of the two. These SSLRs can be calculated for each possible score on the scale in question. Nomograms can then be constructed providing the probability that a given individual is a case depending on their score. While this approach certainly retains more information than a simple cutpoint and may be intuitively appealing, it does not avoid the problem of having to choose a cutpoint in many situations since the SSLRs may still need to be summarised for practical purposes. Furthermore, the SSLR is more useful for diagnostic purposes, while both the GHQ-12 and the MHI-5 are intended as screening tools as opposed to diagnostic tools. The use of depression/anxiety or case finding instruments has limited impact on the recognition, management or outcome of depression/anxiety in primary care [20,21]. As such, a cutpoint on either scale is only appropriate for use in research on populations and would not be suitable for diagnostic purposes.

In the year that wave 9 of the BHPS was carried out (2000), an updated version of the SF-36 was released [5]. The MHI-5 used in the SF-36 version two excludes the response choice "a good bit of the time" as validation studies found that this choice was not consistently ordered in relation to the other categories [22]. However, it has been shown that there is little difference in the performance of the six and five choice response scale [5,17].

## Conclusion

Of the five optimisation methods used in this study, the Youden Index and the (0,1) method are the most suitable for the determination of a generalisable cutpoint, since they are least dependent on the population case prevalence. Both approaches indicate that the best cutpoint to define a case of CMD using the MHI-5 is 76, while for the MCS the Youden Index indicates a cutpoint of 51.7 and the (0,1) method a cutpoint of 52.1. The MHI-5 has the advantage over the GHQ-12 of brevity, consisting of only five multiple choice questions and performs very similarly to the longer MCS. Further validation studies, ideally using a standardised interview schedule and a large population, spanning different countries, are required to confirm our findings.

## Competing interests

The author(s) declare that they have no competing interests.

## Authors' contributions

DLF was responsible for the inception and design of the study. The analysis was performed by MJK, supervised by FDD. MJK drafted the paper. All authors contributed to the intellectual content of the paper as well as offering revisions on all drafts. KL was especially involved in commenting upon the psychiatric component to the paper. All authors read and approved the final manuscript.

## Pre-publication history

The pre-publication history for this paper can be accessed here:


